# Utility of ^18^F-FDOPA PET/CT in the differential diagnosis of canine pheochromocytoma and adrenocortical tumors clinically mimicking pheochromocytoma

**DOI:** 10.1186/s13620-025-00320-4

**Published:** 2025-12-23

**Authors:** Yeon Chae, Hyunglak Son, Su-Hyeon Kim, Hakhyun Kim, Byeong-Teck Kang, Sungin Lee

**Affiliations:** 1 Laboratory of Veterinary Internal Medicine, College of Veterinary Medicine, Chungbuk National University, Cheongju, Chungbuk 28644 Republic of Korea; 2 Department of Veterinary Surgery, College of Veterinary Medicine, Chungbuk National University, Cheongju, Chungbuk 28644 Republic of Korea; 3 Department of Veterinary Surgery, Haemaru Referral Animal Hospital, Seongnam, 13590 Republic of Korea

**Keywords:** Dog, FDOPA, Neuroendocrine tumor, Pheochromocytoma, PET/CT

## Abstract

This study evaluated the utility of 3,4-dihydroxy-6-[18F]-fluoro-l-phenylalanine (^18^F-FDOPA) positron emission tomography/computed tomography (PET/CT) for the differential diagnosis of canine pheochromocytoma. In some cases, conventional diagnostic methods, including hormonal assays and imaging modalities, may have limited ability to differentiate adrenal tumor types in dogs. ^18^F-FDOPA PET/CT, a novel nuclear imaging modality, addresses these limitations by providing functional assessment of catecholamine-producing cells, offering higher accuracy and specificity in detecting pheochromocytomas. In this study, ^18^F-FDOPA PET/CT was effective in localizing a pheochromocytoma, guiding surgical intervention, and aiding clinical decision-making. In cases of adrenocortical tumors clinically mimicking pheochromocytoma, ^18^F-FDOPA PET/CT revealed no significant uptake, facilitating the identification of alternative adrenal pathologies. These results highlight the potential utility of ^18^F-FDOPA PET/CT as a complementary diagnostic tool in cases where conventional methods yield inconclusive results. These findings suggest that using ^18^F-FDOPA PET/CT as a standard diagnostic tool for canine adrenal tumors can enhance diagnostic accuracy and improve treatment planning. Further studies are recommended to validate these findings in larger populations and to establish standardized protocols for veterinary practice.

## Introduction

Pheochromocytoma is a rare neuroendocrine tumor originating from the chromaffin cells of the adrenal medulla [1–5]. Excessive secretion of catecholamines such as adrenaline and noradrenaline leads to episodic or sustained clinical signs, including hypertension, tachycardia, collapse, and restlessness [1–8]. These signs are often vague and intermittent, making antemortem diagnosis difficult. Moreover, pheochromocytomas frequently occur alongside other endocrine or systemic diseases, further obscuring the clinical picture [8, 9]. Adding to diagnostic complexity, certain adrenal or non-adrenal conditions can mimic pheochromocytoma, presenting with episodic hypertension and similar signs in the absence of catecholamine-secreting tumors. These cases are referred to as clinically mimicking pheochromocytoma or pseudopheochromocytoma in human medicine [10]. In veterinary practice, the diagnosis of such conditions is especially challenging due to the nonspecific nature of clinical signs and subjective symptoms such as headaches, palpitations, and sweating. This further emphasizes the need for reliable diagnostic methods to accurately differentiate between true pheochromocytomas and conditions that mimic them. Although the prevalence of pheochromocytoma in dogs is relatively low, its impact on affected animals can be significant and often leads to life-threatening complications. The treatment of choice is surgical resection of the tumor, which can be curative [11–13]. However, the prognosis varies depending on the tumor size, location, and presence of metastasis [14]. Therefore, precise diagnosis and strategic treatment planning are essential.

The diagnosis of pheochromocytomas in dogs typically involves a combination of clinical evaluation, hormonal assessment, and imaging studies. Routine clinical pathology can reveal catecholamine-induced or tumor-associated abnormalities; however, these findings are nonspecific [8, 9]. Hormonal assays to measure catecholamines or their metabolites in plasma or urine samples have been reported in dogs [15–18]. These tests are considered key diagnostic tools for pheochromocytoma; however, their clinical utility may be affected by small sample sizes, which can decrease the reliability of reference ranges and diagnostic thresholds, as well as by possible influences of stress, excessive excitement, exercise, and coexisting conditions, such as impaired renal function [15–19]. Imaging studies are pivotal not only for detecting metastasis and local invasion but also for confirming tumor location and facilitating precise surgical planning. Easily accessible imaging modalities include radiography and ultrasonography. Abdominal radiography reveals a perirenal mass in 26–56% of canine pheochromocytoma cases and can also identify organ displacement due to the mass effect or thoracic metastasis [4, 7, 20]. Abdominal ultrasonography is considered an effective diagnostic tool for evaluating the adrenal area and detecting adrenal masses, with detection rates of 50–83% in canine pheochromocytoma cases [4, 7]. Additionally, ultrasonography can help detect abdominal metastases and local invasion of the kidney or caudal vena cava [4, 7, 20]. Advanced diagnostic tools such as computed tomography (CT), which is currently considered the preferred imaging modality for evaluating pheochromocytoma, and magnetic resonance imaging are useful for diagnosing metastasis and local invasion [3, 8, 20]. Despite their advanced capabilities, these imaging modalities cannot distinguish pheochromocytomas from other adrenal tumors [8, 21]. Ultrasonography-guided fine-needle aspiration is commonly used technique for obtaining cytologic samples from abdominal organs. Adrenal cytology has been shown to accurately distinguish between cortical and medullary origins in adrenal tumors, which can aid clinical decision-making. However, it carries potential risks—including hemorrhage or hypertensive crisis—and is often avoided in clinical practice [8, 22]. Consequently, current methods for accurately differentiating pheochromocytomas may have limitations in veterinary medicine.

Scintigraphy using metaiodobenzylguanidine (MIBG), which has a molecular structure similar to that of norepinephrine, is an advanced diagnostic modality for pheochromocytoma in dogs [23, 24]. Although this technique may offer superior diagnostic capabilities for pheochromocytoma and its metastases compared to conventional imaging methods, it may be less accurate for structural evaluations [25]. In human medicine, 3,4-dihydroxy-6-[18F]-fluoro-l-phenylalanine (^18^F-FDOPA) positron emission tomography/computed tomography (PET/CT) scan has emerged as a highly promising diagnostic tool for neuroendocrine tumors, including pheochromocytomas [26–28]. A fluorinated analog of L-dopa, ^18^F-FDOPA, is taken up by catecholamine-producing cells, allowing for precise imaging of pheochromocytomas [29–31]. Unlike other conventional imaging techniques, ^18^F-FDOPA PET/CT provides functional imaging by highlighting the metabolic activity of the tumor. It offers high diagnostic performance for pheochromocytoma in humans (sensitivity 97%, specificity 94%, and area under the curve of 0.99) and can be particularly useful in cases where conventional imaging is inconclusive [28, 32]. In addition to aiding the accurate localization of primary tumors, ^18^F-FDOPA PET/CT enhances the detection of metastatic disease, facilitating comprehensive disease staging and informed therapeutic decision-making.

This study aimed to explore the potential utility of ^18^F-FDOPA PET/CT in the diagnosis of canine pheochromocytoma. Based on individual clinical observations, the study highlights the possible advantages of ^18^F-FDOPA PET/CT over conventional diagnostic methods and its potential role in guiding clinical decision-making in selected cases.

## Case presentation

### Dog 1: pheochromocytoma

**Fig. 1:**
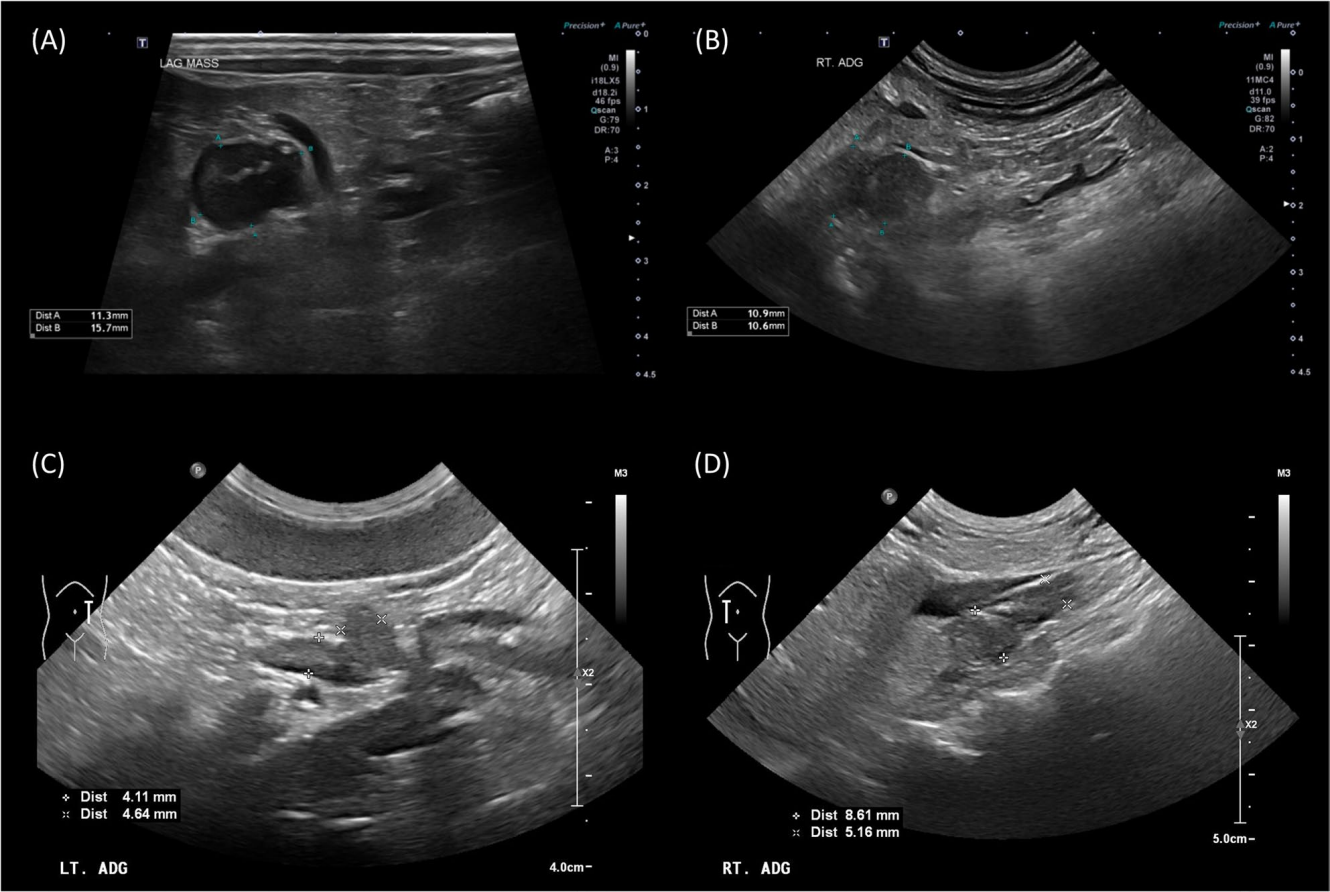
Ultrasonographic findings of adrenal tumors in dogs. Ultrasonographic images of the adrenal glands of dogs with pheochromocytoma (**A**, **B**) and adrenocortical adenoma (**C**, **D**). A well-circumscribed, heterogeneous left adrenal mass measuring 11.3 mm x 15.7 mm with both solid and cystic components compressing the adjacent large vessel. Histopathological examination confirmed the diagnosis of pheochromocytoma (**A**). An irregular, inhomogeneous right adrenal mass measuring up to 10.9 mm (**B**). A normal, crescent-shaped left adrenal gland measuring 4.64 mm at the cranial pole and 4.11 mm at the caudal pole, with a homogenous echotexture (**C**). A right adrenal mass measuring 8.61 mm with a homogeneously hypoechoic echotexture compared to the surrounding liver tissue and an irregular shape. Histopathological examination confirmed the diagnosis of adrenocortical adenoma (**D**)

**Fig. 2:**
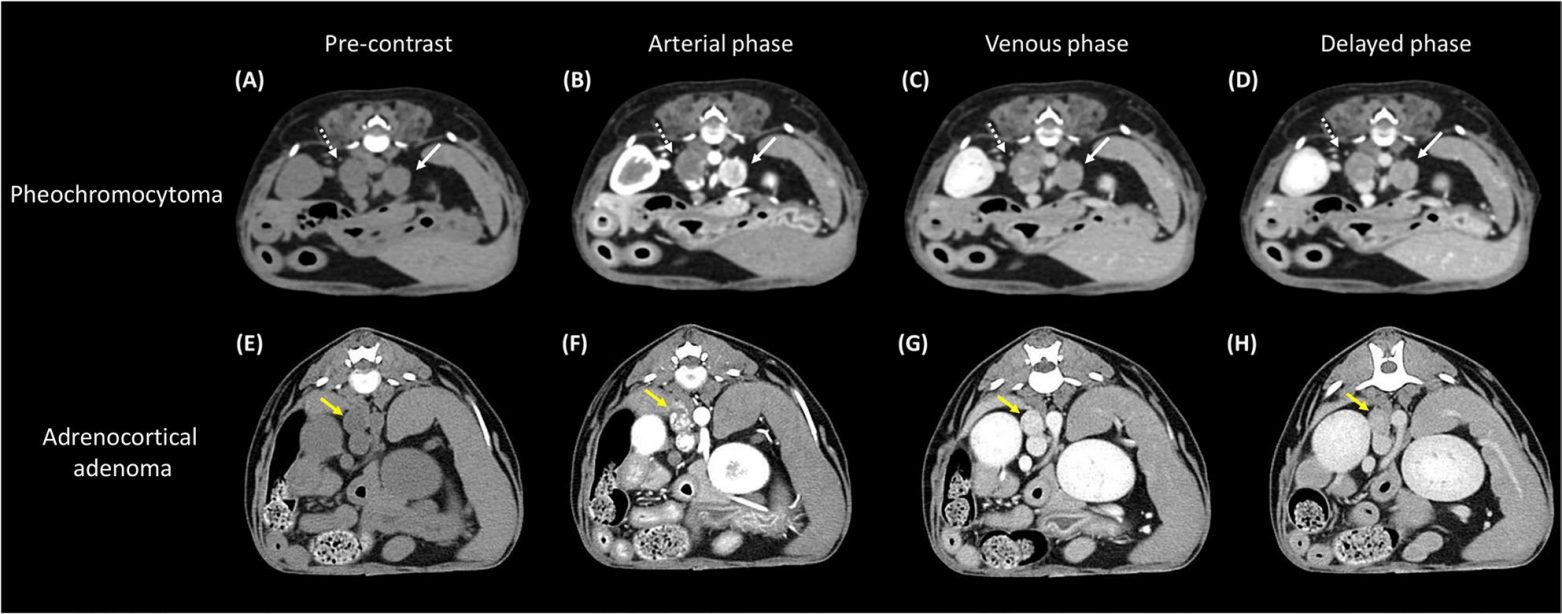
Computed tomographic images of different adrenal tumors in two dogs. Computed tomography images of a pheochromocytoma invading the left adrenal gland of the dog (**A**-**D**). The left (white arrow) and right (white dotted arrow) adrenal glands are enlarged (**A**). Contrast enhancement of the left adrenal gland increases rapidly in the arterial phase (**B**), followed by a gradual decrease in the venous (**C**) and delayed (**D**) phases. The right adrenal gland mildly compresses the caudal vena cava and shows gradual contrast enhancement, reaching its maximum in the venous phase and maintaining this enhancement throughout the delayed phase (**B**-**D**). Computed tomography images of an adrenocortical adenoma in the right adrenal gland (**E**-**H**). The right adrenal gland (yellow arrow) is enlarged and appears homogeneously hypodense in the pre-contrast phase (**E**). In the arterial phase, heterogeneous contrast enhancement with both hyperdense and hypodense regions is observed, followed by a gradual decrease in the venous (**G**) and delayed (**H**) phases

**Fig. 3:**
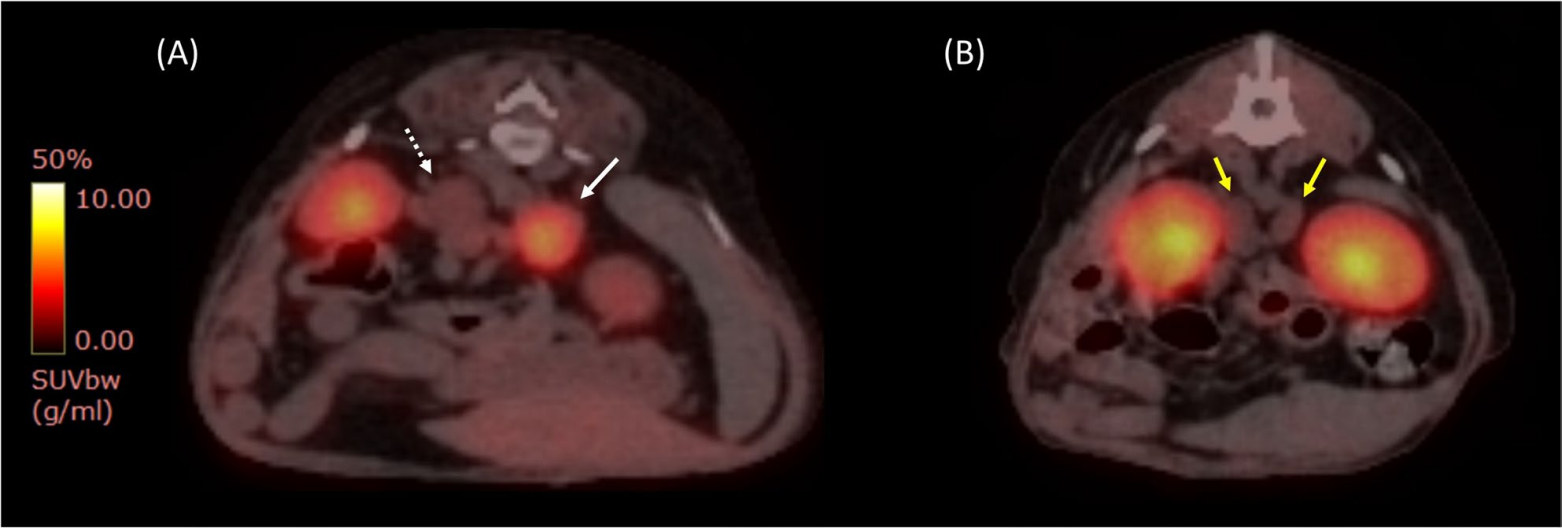
^18^F-FDOPA PET/CT fusion images of adrenal tumors in dogs. ^18^F-FDOPA PET/CT fusion image showing intense tracer uptake in the left adrenal pheochromocytoma (white arrow). The lesion demonstrates markedly increased ^18^F-FDOPA accumulation compared to the physiological uptake in the contralateral adrenal mass (white dotted arrow), indicating a catecholamine-producing tumor. The SUVmax of the pheochromocytoma was 5.34, while that of the contralateral adrenal mass was 1.80 (**A**). ^18^F-FDOPA PET/CT fusion image of a dog with an adrenocortical adenoma. Both adrenal glands (yellow arrows) demonstrate minimal tracer uptake (left, SUVmax 1.23; right, SUVmax 1.28), suggesting physiological metabolic activity and reduced catecholamine synthesis compared to pheochromocytoma (**B**). ^18^F-FDOPA, 3,4-dihydroxy-6-[18F]-fluoro-l-phenylalanine; SUVmax, maximum standardized uptake value

**Fig. 4:**
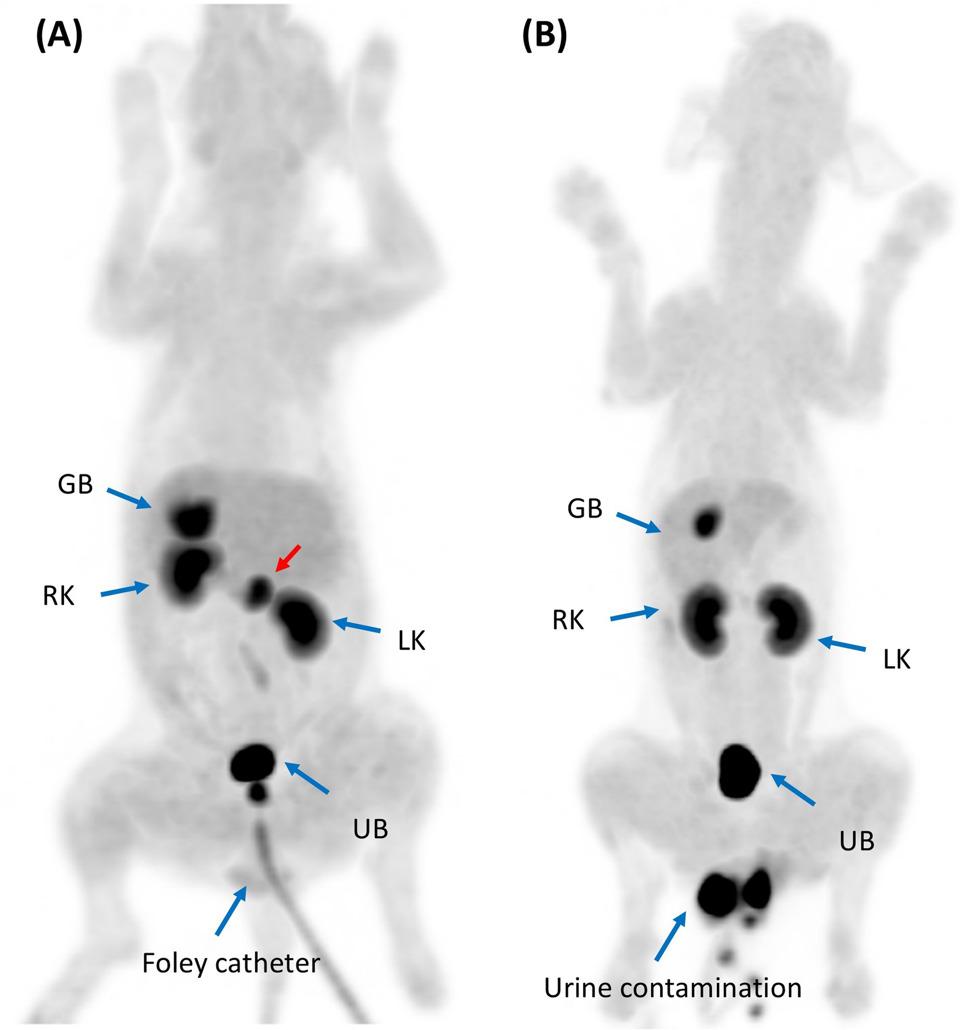
Maximum intensity projection (MIP) of the whole body. Dorsal view of MIP images of the dog with pheochromocytoma (**A**) and adrenocortical adenoma (**B**). The MIP image shows a solitary left adrenal pheochromocytoma (red arrow) with high tracer avidity. The lesion demonstrates markedly increased ^18^F-FDOPA uptake compared to the physiological distribution in other organs. ^18^F-FDOPA, 3,4-dihydroxy-6-[18F]-fluoro-l-phenylalanine; GB, gallbladder; LK, left kidney; RK, right kidney; UB, urinary bladder

**Fig. 5:**
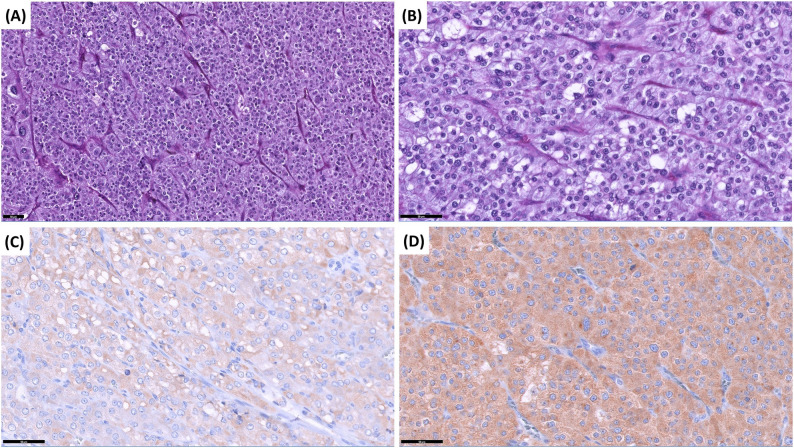
Histopathology of pheochromocytoma. Histopathological and immunohistochemical features of pheochromocytoma. The tumor shows a characteristic Zellballen pattern, with nests of chief cells surrounded by sustentacular cells (**A**). The neoplastic cells have distinct cellular borders, with pale eosinophilic to amphophilic, finely granular cytoplasm. Nuclei are round or ovoid, with one or more nucleoli (**B**). Immunohistochemistry shows moderate diffuse immunostaining for synaptophysin (**C**) and strong diffuse immunostaining for chromogranin A (**D**), supporting the diagnosis of pheochromocytoma. Scale bars: 50 μm (**A**-**D**)

A 13-year-old spayed female Maltese was referred for ^18^F-FDOPA PET/CT after being diagnosed with hypercortisolism and pheochromocytoma at a local veterinary clinic. During a routine health examination at a primary veterinary care facility, enlarged bilateral adrenal glands were detected. Subsequently, the dog was referred to a secondary veterinary care facility for the differential diagnosis of bilateral adrenomegaly. The reported clinical signs included panting, polyuria, and polydipsia. Upon history taking, the dog had no concurrent diseases or medications and was maintained on a commercial diet. On physical examination, the dog exhibited an elevated blood pressure of 160 mmHg, but no other significant abnormalities were observed. Blood examination revealed an elevated alanine aminotransferase (ALT) level of 133.73 U/L (reference interval [RI]: 19–70 U/L) and an increased alkaline phosphatase (ALP) level of 250 U/L (RI: 15–127 U/L). The results of the complete blood count (CBC) were unremarkable. Hormonal assessments included an adrenocorticotropic hormone (ACTH) stimulation test, which revealed a baseline cortisol level of 8.8 µg/dL and a post-1-hour cortisol level of 30 µg/dL, consistent with hypercortisolism [33]. A low-dose dexamethasone suppression test (LDDST) was also performed, resulting in a baseline cortisol of 3.4 µg/dL, with post-4-hour and post-8-hour cortisol levels of 3.2 µg/dL and 2.9 µg/dL, respectively, leading to the diagnosis of hypercortisolism based on these non-suppressed results [33]. The urine normetanephrine-to-creatinine ratio (uNMN/Cr) was 794.4, and the urine metanephrine-to-creatinine ratio (uMN/Cr) was 858.1, as measured using liquid chromatography-tandem mass spectrometry. The uNMN/Cr value was >364, a cut-off previously proposed as four times the highest value in normal, which is highly suggestive of a pheochromocytoma [15]. Abdominal ultrasonography revealed two adrenal masses. The left adrenal mass measured 11.3 × 15.7 mm with suspected vascular invasion. The right adrenal mass was 13 × 15.11 mm and appeared to compress the caudal vena cava. (Figure 1A and B). Based on these findings, a surgical removal plan was recommended considering the potential malignancy of the pheochromocytoma. However, accurate differentiation of the affected adrenal gland, whether unilateral or bilateral, was impossible owing to the presence of bilateral adrenal masses. On computed tomography, a left adrenal mass (11 × 16 mm) demonstrated rapid contrast enhancement. It displayed 56 Hounsfield units (HU) in the pre-contrast phase, which rose sharply to 247 HU in the arterial phase, followed by a decline to 130 HU in the venous phase and 114 HU in the delayed phase. Invasion of the left phrenico-abdominal vein was suspected. The right adrenal gland, measuring 13 × 15 mm with a pre-contrast HU of 47, showed gradual enhancement, peaking at 122 HU in the venous phase and maintaining this level through the delayed phase, with mild compression and suspected invasion into the caudal vena cava (Fig. 2A-D). Based on these findings, a pheochromocytoma was suspected in the left adrenal gland; however, it was not possible to determine the tumor type or functional status of the right adrenal gland [34]. Additionally, no remarkable findings were observed in abdominal organs or lymph nodes. Thoracic and brain CT was performed to screen for possible metastasis and pituitary abnormalities, respectively, and no suspicious lesions were identified. Therefore, ^18^F-FDOPA PET/CT was performed for the localization of pheochromocytoma, as well as for the evaluation of metastasis and staging. ^18^F-FDOPA PET/CT scan was conducted following a 12-hour fasting period to optimize tracer uptake and general anesthesia. ^18^F-FDOPA (3.5 MBq/kg) was administered intravenously into the cephalic vein. General anesthesia was induced 45 min after ^18^F-FDOPA administration using intravenous propofol (4 mg/kg; Provive, Myungmoon Pharm. Co., Ltd., Seoul, South Korea) and maintained with 2.5% isoflurane (Terrell, Piramal Critical Care, Inc., Bethlehem, PA, USA). Low-dose CT images were acquired both before and after intravenous injection of iohexol (2 mL/kg) (Omnipaque 350; GE Healthcare) to enhance imaging quality. Subsequently, PET imaging was performed 60 min after the radiotracer injection using a Discovery-STE PET/CT scanner (General Electric Medical Systems, Waukesha, WI, USA). The whole-body scan lasted 3 min per bed position. PET images were analyzed using a commercial program (OsiriX MD v10.0; Pixmeo Sarl, Geneva, Switzerland). Regions of interest (ROI) were drawn manually on the PET/CT fusion images, and the metabolic activity within the ROIs was converted to a standardized uptake value (SUV), which was calculated as follows: SUV = average tissue concentration of ^18^F-FDOPA (MBq/ml)/injected dose (MBq) per body weight (g). The maximum standardized uptake value (SUVmax) of the right adrenal gland was 1.80, while the left adrenal gland exhibited increased ^18^F-FDOPA avidity, with an SUVmax of 5.34 (Fig. 3A); an SUVmax cut-off of 4.1 (sensitivity 93%, specificity 85%) has been proposed in human medicine for identifying pheochromocytoma [35]. Physiological excretion was observed in the gallbladder, kidneys, and bladder. No significant abnormalities were observed in other organs, including the pituitary gland (Table 1; Fig. 4A). Therefore, a diagnosis of unilateral left adrenal pheochromocytoma without metastasis was made [35]. Based on these findings, a left adrenalectomy was performed, and pheochromocytoma was confirmed through histopathological examination and immunohistochemistry (Fig. 5A-D). After the surgery, the dog recovered without any complications. After one month of the surgery, the LDDST indicated a baseline cortisol level of 3.8 µg/dL, with post-4-hour and post-8-hour cortisol levels of 5.7 µg/dL and 5.3 µg/dL, respectively, suggesting persistent hypercortisolism; treatment with trilostane was initiated [33]. At one month of treatment, the post-trilostane 3-hour cortisol concentration was 2.6 µg/dL, within the ideal control range (1.45–5.0 µg/dL) [36]. At that time, uNMN/Cr and uMN/Cr decreased to 115.7 and 39.3, respectively, indicating improvement from pretreatment levels and suggesting remission of pheochromocytoma. The dog was monitored regularly using post-trilostane 3-hour cortisol measurements, which remained within the ideal range, including a value of 2.3 µg/dL at 7 months. Liver enzyme elevations also normalized during follow-up, and no clinical signs of hypercortisolism were observed, indicating successful long-term disease control.

**Table 1:** Physiologic distribution of ^18^F-FDOPA in dogs with adrenal tumors

	Dog 1 (Pheochromocytoma)		Dog 2 (Adrenocortical adenoma)	
	SUVmax	SUVmean	SUVmax	SUVmean
Adrenal gland				
Pheochromocytoma	5.34	3.86	-	-
Adrenocortical adeoma	1.80	1.53	1.23	1.16
Presumed normal	-	-	1.28	1.17
Liver	1.64	1.49	1.69	1.45
Spleen	0.69	0.56	0.70	0.52
Pancreas	0.93	0.90	0.95	0.83
Stomach	1.25	1.12	1.28	1.14
Thyroid	0.62	0.58	0.86	0.80
Myocardium	1.04	0.95	1.10	0.99
Kidney (Parenchyma)	4.20	3.28	5.04	4.12
Kidney (Pelvis)	12.31	8.59	9.45	7.64
Urinary bladder	33.7	12.28	153.73	46.52
Gall bladder neck	6.10	2.53	8.30	5.57

SUVmax, maximum standardized uptake value

SUVmean, mean standardized uptake value

### Dog 2: adrenocortical tumor clinically mimicking pheochromocytoma

**Fig. 6:**
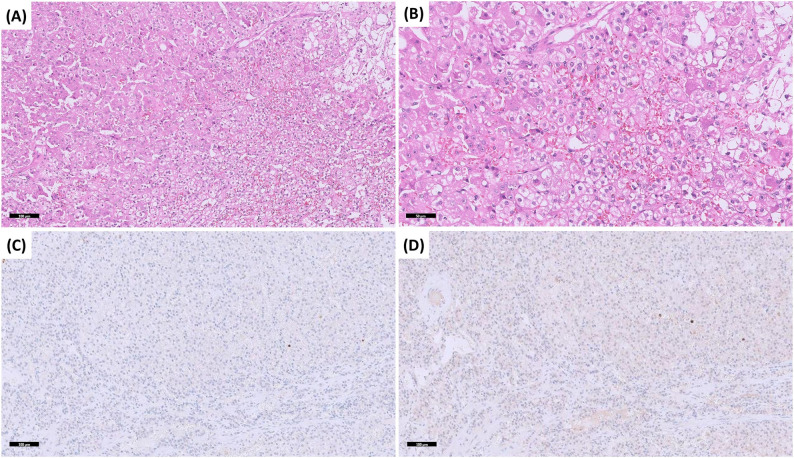
Histopathology of adrenocortical adenoma. Histopathological and immunohistochemical features of adrenocortical adenoma. The histomorphology of the neoplastic cells is most consistent with abundant vacuolated cytoplasm (**A**) without discernible chromaffin granules, a characteristic feature of pheochromocytoma with a rich vascular network (**B**). Negative staining for synaptophysin (**C**) and chromogranin A (**D**) further supports the diagnosis of an adrenocortical adenoma and excludes pheochromocytoma. Scale bars: 100 μm (**A**, **C**, **D**) and 50 μm (**B**)

An 11-year-old intact female Shih Tzu was referred for treatment of a liver mass identified during a routine health examination at a primary veterinary clinic, which included physical examination, blood tests, radiography, and abdominal ultrasonography. Upon history taking, the dog had no other diagnosed diseases or prescribed medications and was maintained on a commercial diet. The dog presented with no discernible clinical signs. Physical examination revealed no significant findings. Blood analysis revealed mild thrombocytosis, with a platelet count of 571 × 10³/µL (RI: 148–484 × 10³/µL) on the CBC. Serum chemistry revealed elevated liver enzyme levels, including ALT at 731 IU/L (RI: 21–102 IU/L), ALP at 2154 IU/L (RI: 29–97 IU/L), and Gamma-Glutamyl Transferase at 43 IU/L (RI: 1–10 IU/L). The electrolytes, including the sodium/potassium ratio, remained within normal limits. Abdominal ultrasonography was performed to evaluate the liver mass and screen for potential metastasis. A single, ill-defined, amorphous mass lesion was identified in the region of the left lateral liver lobe, measuring approximately 46.4 × 33.1 mm (length × height). The lesion had irregular margins and showed a moderately heterogeneous echotexture, characterized by iso- to hypoechoic areas relative to the surrounding liver parenchyma, with internal anechoic cavitary regions. Mild intralesional vascular flow was detected on color doppler imaging. Enlargement of the right adrenal gland was incidentally detected, while the left adrenal gland appeared normal (Fig. 1C and D). An approximately 9 mm mass was observed at the cranial pole of the right adrenal gland, which was in contact with a mass originating from the caudate lobe of the liver (Fig. 1D). The adrenal mass appeared homogeneously hypoechoic compared to the surrounding liver tissue and exhibited irregular margins. No significant blood flow was detected within the mass on color Doppler, with no signs of invasion of the surrounding structures. The left adrenal gland appeared normal in size and appearance (Fig. 1C). The right adrenal gland enlargement was incidentally identified, prompting an adrenal function assessment to differentiate concurrent endocrine diseases or malignancies prior to liver surgery. Hormonal assessments were conducted to evaluate cortical and medullary adrenal function. The results of LDDST were consistent with hypercortisolism, showing a baseline cortisol level of 3.37 µg/dL, a post-4-hour cortisol level of 2.24 µg/dL, and a post-8-hour cortisol level of 1.42 µg/dL [33]. The urinary NMN/Cr was 158, and the uMN/Cr was 123.5, both within normal reference ranges [15]. Consequently, hormonal assessment was consistent with hypercortisolism, and pheochromocytoma was considered improbable. Therefore, left hilar liver lobectomy was performed, and concurrent adrenalectomy was initially considered. During surgery, sudden hypertension reaching a systolic blood pressure of 240 mmHg was observed upon manipulation of the organs surrounding the right adrenal gland. Given these intraoperative findings, adrenalectomy was deferred for further evaluation and preoperative stabilization. Despite aggressive postoperative pain management, intermittent hypertension—ranging between 180 and 220 mmHg—persisted throughout the hospitalization period. Postoperative medications included remifentanil infusion for 3 days for pain management, followed by a fentanyl patch for 5 days. Cefazolin was administered for 3 days, along with famotidine for gastrointestinal protection. Hepatoprotective agents, including ursodeoxycholic acid and S-adenosylmethionine, were continued after discharge. No drugs known to interfere with urinary metanephrine levels were used during this period. Histopathological examination confirmed well-differentiated hepatocellular carcinoma with minimal cellular atypia, no bile ducts, and no vascular invasion. Owing to the persistence of hypertension one month after the surgery, a re-evaluation of adrenal function was indicated. Urine catecholamine metabolite analysis revealed elevated uNMN/Cr of 530.4 and uMN/Cr of 319.3, strongly indicating the presence of a potential pheochromocytoma [15]. On CT examination, the right adrenal gland of the dog was enlarged, measuring 11 mm at the cranial pole and 5.71 mm at the caudal pole, showing homogeneously hypodense enhancement in the pre-contrast phase (HU 45) with heterogeneous contrast enhancement (maximum 200 HU) in the arterial phase. Contrast enhancement gradually decreased during the venous and delayed phases (Fig. 2E-H). The left adrenal gland measured 5.61 mm (cranial pole) and 5.21 mm (caudal pole), with an attenuation of 25 HU in the pre-contrast phase and a maximum of 80 HU in the post-contrast phases, without structural abnormalities. Thoracic and abdominal CT revealed no additional abnormalities. To differentiate pheochromocytoma, ^18^F-FDOPA PET/CT was conducted according to the previously mentioned protocols; however, no significant ^18^F-FDOPA uptake was observed in the right adrenal gland. The SUVmax of the right adrenal gland was measured at 1.23, while the left adrenal gland exhibited an SUVmax of 1.28 (Fig. 3B). Physiological excretion was observed in the gallbladder, kidneys, and bladder; however, no significant uptake was observed in other tissues, including the pituitary gland (Table 1; Fig. 4B). The ^18^F-FDOPA PET/CT results indicated a low likelihood of pheochromocytoma in the right adrenal gland. These findings effectively ruled out a functionally active pheochromocytoma, supporting the surgical plan without the need for additional preoperative management. Given the results, a right adrenalectomy was performed due to the possibility of metastasis from hepatocellular carcinoma or other right adrenal gland malignancies, such as adrenocortical carcinoma, which could not be excluded. In addition, adrenal-dependent hyperadrenocortisolism, as suggested by endocrinologic evaluation, was also considered a potential cause of the persistent hypertension and contributed to the decision for surgical intervention. During adrenal manipulation in surgery, the systolic blood pressure increased from 120 to 245 mmHg, fluctuating between 180 and 245 mmHg, and was controlled with nitroprusside and esmolol administration. After the surgery, intermittent hypertension was observed during hospitalization; however, the dog recovered without significant complications and was subsequently discharged. Histopathological examination confirmed adrenocortical adenoma, with negative immunohistochemical staining for medullary markers (Fig. 6A-D). The absence of a pheochromocytoma was confirmed, suggesting that the clinical presentation of hypertension could be attributed to adrenocortical pathology or other concurrent factors. Three months after right adrenal gland adrenalectomy, the dog continued to show intermittent hypertension upon presentation, which was well managed without notable complications or metastasis of hepatocellular carcinoma. Follow-up adrenal function tests revealed a urinary cortisol-to-creatinine ratio of 26.01 × 10^− 6^ (< 40 × 10^− 6^), with uNMN/Cr and uMN/Cr values of 262.3 and 72.1, respectively [37]. These findings indicated the resolution of hypercortisolism, but the persistence of hypertension suggested the potential involvement of other systemic factors that remain under observation.

## Discussion and conclusion

Conventional diagnostic methods, such as hormonal assays and imaging techniques, may have certain limitations in accurately and specifically differentiating between various types of adrenal tumors, particularly pheochromocytomas. Although hormonal assessment provides insights into both cortical and medullary adrenal function, it cannot distinguish between benign and malignant adrenal tumors or determine the affected gland in cases of bilateral disease. Imaging modalities, such as ultrasonography and CT, are effective in detecting malignancy indicators, including irregularly shaped masses or increased vascularity. However, these diagnostic modalities are limited in their ability to differentiate between the various histological types of adrenal tumors. Furthermore, invasive procedures such as adrenal biopsies involve significant risks and are not always feasible. The dogs in this study also presented with ambiguous clinical signs, and hormonal assessments confirmed the presence of both hypercortisolism and pheochromocytoma. However, conventional imaging modalities were unable to specifically identify adrenal tumors associated with pheochromocytoma. ^18^F-FDOPA PET/CT, a novel nuclear medicine diagnostic modality, facilitates the differential diagnosis of pheochromocytomas by detecting increased abnormal catecholamine synthesis in the adrenal medulla. This diagnostic modality addresses the limitations of the conventional methods and provides valuable information to assist in treatment planning and prognostic evaluation.

To address the shortcomings of traditional diagnostic approaches for pheochromocytomas, there is growing interest in the use of nuclear imaging modalities as possible new gold standards for detection and evaluation. Although ^123^I-MIBG and ^131^I-MIBG are traditional nuclear imaging techniques used to detect pheochromocytomas in veterinary practice, their widespread application is still limited [23, 24]. MIBG scanning utilizes the structural similarity of MIBG to norepinephrine, which enables the localization of adrenal, ectopic, and disseminated pheochromocytomas in humans [38]. However, MIBG scanning using ^123^I- or ^131^I-labeled MIBG can be time-consuming and often requires a waiting period of 24–48 h post-injection for optimal imaging [23, 24, 39, 40]. Moreover, pre-treatment for thyroid blocking is necessary to prevent iodine uptake by the thyroid gland [41]. In contrast to this prolonged process, ^18^F-FDOPA PET/CT enables diagnosis within 45–90 min without the need for pretreatment. Additionally, ^18^F-FDOPA PET and PET/CT are highly sensitive and specific tools for accurate diagnosis and localization of pheochromocytomas [28]. A comparative study of pheochromocytomas in humans found that ^18^F-FDOPA PET achieves a higher patient-based sensitivity than that of ^123^I-MIBG scintigraphy (90% vs. 65%) [42]. Similarly, lesion-based sensitivities were higher with ^18^F-FDOPA PET than with ^123^I-MIBG scintigraphy (73% vs. 48%) [42]. Furthermore, the reliance on planar imaging in ^123^I-MIBG scans limits their diagnostic value, especially because most studies do not incorporate SPECT/CT, a technique known to improve diagnostic accuracy. Despite its advantages, the use of SPECT/CT in veterinary medicine remains limited.

In this study, the dog (Dog 1) presented with concurrent pheochromocytoma and hypercortisolism. Pheochromocytoma, a tumor of chromaffin cells, can occur alongside conditions such as Cushing’s syndrome, highlighting the potential for overlapping pathophysiologies. In some cases, pheochromocytomas may secrete ectopic ACTH or corticotropin-releasing hormone, which can stimulate the adrenal cortex, induce hypercortisolism, and lead to Cushing’s syndrome [43–45]. Alternatively, the paracrine effects of ACTH produced locally by pheochromocytomas on the ipsilateral adrenal cortex may lead to apparent ACTH-independent Cushing’s syndrome [43, 44, 46]. Hyperplasia of the adjacent adrenal cortex observed in some cases supports this paracrine mechanism [43, 44, 46]. Additionally, the chronic stimulation of the adrenal cortex by catecholamines from pheochromocytomas may contribute to cortisol overproduction [43, 44, 46]. If hypercortisolism were secondary to pheochromocytoma, cortisol levels would have normalized following unilateral adrenalectomy. However, in this dog, hypercortisolism persisted postoperatively, suggesting an alternative pathophysiology of hypercortisolism. Therefore, it is more appropriate to suspect the contralateral adrenal mass as the source of hypercortisolism rather than attributing it to pheochromocytoma. According to previous human studies, the concurrent occurrence of pheochromocytoma and Cushing’s syndrome is rare [47]. In dogs, although concurrent endocrine neoplasia is also uncommon, its prevalence is reported to be higher than in humans. The most frequently observed combinations involve neoplasms of the pituitary gland, adrenal medulla, and/or adrenal cortex, indicating a distinct pattern from that seen in human patients [48, 49]. Given the shared clinical features of these two diseases, pheochromocytoma could be underdiagnosed, particularly because of the relative inaccuracy of hormonal analysis under various conditions. In human medicine, pheochromocytoma is frequently underdiagnosed and is often identified incidentally or only during autopsies [50, 51]. Similar to findings in humans, pheochromocytomas in dogs are frequently discovered incidentally, with 48% of cases identified unexpectedly during necropsy or surgery, highlighting the potential for underdiagnosis in veterinary practice [4, 7]. Moreover, a previous study in dogs unexpectedly identified pheochromocytoma in three of six dogs with hyperadrenocorticism at autopsy, suggesting that concurrent pheochromocytoma could be underdiagnosed [52]. Under these circumstances, ^18^F-FDOPA PET/CT offers distinct advantages by enabling the direct visualization of catecholamine-producing tissue in the adrenal medulla, thereby allowing for a more precise diagnosis of pheochromocytoma—independent of adrenocortical activity such as concurrent hypercortisolism. In differentiating adrenomegaly cases, expanding ^18^F-FDOPA PET/CT utilization in veterinary medicine could significantly improve diagnostic accuracy and facilitate a more precise assessment of adrenal tumors, such as pheochromocytoma. In both cases presented in this study, concurrent involvement of the adrenal cortex and medulla was initially suspected based on hormonal and imaging findings—a diagnostic context in which pheochromocytoma may be underdiagnosed or diagnosis delayed due to overlapping clinical features with hyperadrenocorticism. The use of ¹⁸F-FDOPA PET/CT enabled accurate diagnosis in one case and definitive exclusion of pheochromocytoma in the other, with findings confirmed by histopathology. Early and appropriate intervention based on functional imaging findings may significantly improve clinical decision-making in such scenarios. Further studies in larger populations are required to evaluate their broader clinical utility and significance.

This study emphasizes the diagnostic challenges associated with identifying pheochromocytomas in canine patients, particularly in cases with overlapping clinical features. While the other dog (Dog 2) presented with sustained hypertension and elevated urine catecholamine metabolites—exceeding four times the upper normal limit—suggestive of pheochromocytoma, histopathology revealed a different adrenal tumor type. This underscores the importance of considering conditions clinically mimicking pheochromocytoma as a differential diagnosis in dogs with suspected pheochromocytoma and the potential value of advanced imaging techniques such as ^18^F-FDOPA PET/CT. In this case, the initial suspicion of pheochromocytoma was based on the presence of hypertension, elevated urine catecholamine metabolite levels, and an adrenal mass on imaging. However, the final diagnosis of cortical adenoma demonstrates that other adrenal tumors can mimic the clinical presentation of pheochromocytoma, consistent with previous studies [53–55]. Increased catecholamine levels are often associated with hyperadrenocorticism. The adrenal medulla, which produces catecholamines, is influenced by blood supply from the adrenal cortex, where cortisol can stimulate catecholamine synthesis [56]. In this dog diagnosed with adrenocortical adenoma, high urine catecholamine metabolites could be influenced by cortisol, which stimulates catecholamine synthesis; however, no evidence of adrenal medullary hyperplasia or neoplasia was observed. These observations highlight the need for cautious interpretation of hormonal assays given the variability in physiological and environmental factors. ^18^F-FDOPA PET/CT, by contrast, directly visualizes pathological catecholamine overproduction in the adrenal medulla, thereby offering a reliable, minimally invasive option for diagnosing pheochromocytoma without the limitations of hormone-based tests. Integrating ^18^F-FDOPA PET/CT can thus enhance clinical accuracy in suspected pheochromocytoma cases. However, given the intermittent hypertension and mildly elevated urinary catecholamine metabolites persisting after adrenalectomy, alternative etiologies should be considered.

Other potential causes of clinically mimicking pheochromocytoma, as reported in humans, include medication-induced effects, stress-related factors, and conditions such as obstructive sleep apnea [57]. These factors can lead to false-positive hormonal assay results for catecholamines and their metabolites. Previous studies on human pheochromocytomas have identified common causes of false positives, including the use of prescribed medications such as tricyclic antidepressants and serotonin-norepinephrine reuptake inhibitors, high catecholamine diets, or conditions associated with increased sympathetic activity, such as heart failure, renal failure, and hypoglycemia [58–60]. However, these factors were not considered likely in this case, as the dog was maintained on a commercial diet, had no underlying diseases, and was not prescribed any medications. Other factors that have been previously reported in both human and veterinary medicine include physiological stress [58–61]. Stress and anxiety during sample collection, as observed in this excited dog, may also have increased the catecholamine levels. In humans, patients with obstructive sleep apnea are more likely to have false-positive urinary metanephrines because of increased nocturnal sympathetic activity [58–63]. This dog was a Shih Tzu, a breed known for its predisposition to brachycephalic obstructive airway syndrome, which serves as a naturally occurring animal model for obstructive sleep apnea in humans [64]. Therefore, medical conditions, such as brachycephalic obstructive airway syndrome in dogs, can lead to elevated catecholamine levels and potentially false-positive results in the diagnosis of pheochromocytoma. Our findings align with those of previous reports, indicating that adrenocortical tumors clinically mimicking pheochromocytoma can occur under various circumstances in humans, posing a diagnostic challenge for veterinarians. The discrepancy between clinical suspicion and histopathological diagnosis in this case reinforces the concept that conditions clinically mimicking pheochromocytoma should be considered in the differential diagnosis of dogs presenting with adrenal masses and hypertension, even when clinical findings strongly suggest pheochromocytoma. While non-invasive conventional diagnostics can provide valuable information, a definitive diagnosis often requires histopathological examination of the adrenal tissue. The integration of functional imaging techniques, such as ^18^F-FDOPA PET/CT, which has demonstrated high sensitivity and specificity in human studies, could potentially improve the accuracy of diagnosis in veterinary patients with suspected pheochromocytoma or clinically mimicking conditions.

In conclusion, ^18^F-FDOPA PET/CT offers promising advantages for the accurate diagnosis of pheochromocytoma, which can often be underdiagnosed because of overlapping clinical features with hyperadrenocorticism and the limitations of conventional diagnostics. By providing a minimally invasive method for identifying catecholamine-producing tissues, ^18^F-FDOPA PET/CT enhances diagnostic precision and supports clinical decision-making. Although further studies are needed to validate its broader clinical utility, our findings highlight its potential role in veterinary patients with suspected pheochromocytoma or clinically mimicking conditions.

## Data Availability

The data generated or analyzed during this case study are included in this published article. Additional information is available from the corresponding author upon reasonable request.

## Abbreviations

^18^F-FDOPA: 3,4-dihydroxy-6-[18F]-fluoro-l-phenylalanine
ACTH: Adrenocorticotropic hormone
ALP: Alkaline phosphatase
ALT: Alanine aminotransferase
CBC: Complete blood count
HU: Hounsfield units
LDDST: Low-dose dexamethasone suppression test
MIBG: Metaiodobenzylguanidine
PET/CT: Positron emission tomography/computed tomography
SUV: Standardized uptake value
SUVmax: Maximum standardized uptake value
uNMN/Cr: Urine normetanephrine-to-creatinine ratio
uMN/Cr: Urine metanephrine-to-creatinine ratio
